# Deep Sequencing Analysis of RNAs from Citrus Plants Grown in a Citrus Sudden Death-Affected Area Reveals Diverse Known and Putative Novel Viruses

**DOI:** 10.3390/v9040092

**Published:** 2017-04-24

**Authors:** Emilyn E. Matsumura, Helvécio D. Coletta-Filho, Shahideh Nouri, Bryce W. Falk, Luca Nerva, Tiago S. Oliveira, Silvia O. Dorta, Marcos A. Machado

**Affiliations:** 1 Instituto de Biociências da Universidade Estadual Paulista (Unesp), Botucatu, SP, 18615-689, Brazil; emilyn.matsumura@gmail.com; 2 Centro de Citricultura Sylvio Moreira, Instituto Agronômico de Campinas, Cordeirópolis, SP, 13490-970, Brazil; helvecio@centrodecitricultura.br (H.D.C.-F.); tsvoliveira@gmail.com (T.S.O.); dorta.silvia@gmail.com (S.O.D.); 3 Department of Plant Pathology, University of California, Davis, CA 9561608751, USA; shahidehnr@gmail.com (S.N.); bwfalk@ucdavis.edu (B.W.F.); 4 Institute for Sustainable Plant Protection (IPSP), CNR, Turin, 10135, Italy; luca.nerva@unito.it

**Keywords:** citrus sudden death, CSDaV, CTV, plant viruses, high-throughput sequencing

## Abstract

Citrus sudden death (CSD) has caused the death of approximately four million orange trees in a very important citrus region in Brazil. Although its etiology is still not completely clear, symptoms and distribution of affected plants indicate a viral disease. In a search for viruses associated with CSD, we have performed a comparative high-throughput sequencing analysis of the transcriptome and small RNAs from CSD-symptomatic and -asymptomatic plants using the Illumina platform. The data revealed mixed infections that included Citrus tristeza virus (CTV) as the most predominant virus, followed by the Citrus sudden death-associated virus (CSDaV), Citrus endogenous pararetrovirus (CitPRV) and two putative novel viruses tentatively named Citrus jingmen-like virus (CJLV), and Citrus virga-like virus (CVLV). The deep sequencing analyses were sensitive enough to differentiate two genotypes of both viruses previously associated with CSD-affected plants: CTV and CSDaV. Our data also showed a putative association of the CSD-symptomatic plants with a specific CSDaV genotype and a likely association with CitPRV as well, whereas the two putative novel viruses showed to be more associated with CSD-asymptomatic plants. This is the first high-throughput sequencing-based study of the viral sequences present in CSD-affected citrus plants, and generated valuable information for further CSD studies.

## 1. Introduction

Citrus sudden death (CSD) is a disease that was first detected in 1999 in citrus groves located in the municipality of Comendador Gomes (southwestern Minas Gerais State), Brazil [1]. At that time, CSD was found to affect only plants of sweet orange (*Citrus sinensis* L. Osbeck) grafted on Rangpur lime rootstock (*Citrus limonia* L. Osb.), a very important drought-resistant rootstock used in Brazil [1]. However, CSD quickly spread into the northern part of São Paulo State and, since then, has caused the death of four million orange trees [2,3]. The symptoms of CSD are characterized by a general decline, including pale green coloration of the leaves, overall defoliation, death of the roots and presence of a characteristic yellow stain in the rootstock bark [4]. Later, CSD-symptoms were also detected in sweet oranges grafted on the other rootstocks, such as *Citrus volkameriana* [3], *Citrus jambiri* and *Citrus pennivisiculata* Lush [5].

The main challenge in studying CSD is that the etiology has not been definitively determined, even after seventeen years from its first detection. Similarities with quick-decline form of citrus tristeza disease, mainly on the symptoms and distribution of the CSD-affected plants, have led previous works to hypothesize that a new variant of Citrus tristeza virus (CTV), a member of the family *Closteroviridae* and one of the most economically important citrus viruses, might be associated in developing CSD symptoms [2,3,6,7]. However, several attempts in trying to identify an isolate or a new variant of CTV associated with CSD, have failed [2,6,8,9]. Conventional shotgun sequencing (which did not make use of the next-generation sequencing (NGS) technology) of complementary DNA (cDNA) derived from double-stranded RNA (dsRNA) isolated from plants showing CSD symptoms was able to identify a novel virus from the family *Tymoviridae* [9]. It was suggested that this new virus was likely to be associated with CSD and was named Citrus sudden death-associated virus (CSDaV). However, the role of this virus in CSD-affected plants is still not completely clear. The conventional shotgun sequencing approach, after the low-quality reads were removed, generated a low number of valid reads [9], which made it difficult to study the frequency of detected viruses, to differentiate virus isolates and to discover novel viruses that might be involved with CSD disease. In addition, conventional approaches for virus detection require prior knowledge of genome sequences [10], thereby allowing for identification only of specific known viruses, and thus is not suitable to study the virome within plants [10,11].

NGS has been widely and successfully used for improved detection and characterization of known and novel viruses in infected plant hosts [11,12]. Deep sequencing of the transcriptome (RNA-seq) and small RNAs (sRNAs) have been shown to be a promising and powerful approach in detecting both RNA and DNA viruses [10,13]. Consequently, this approach can be used to better understand plant diseases, especially when the viral etiology is unknown, as well as to explore plant virus–host interactions [12,14].

In order to identify putative viruses associated with citrus plants affected by CSD, and compare the frequency and diversity of viruses between CSD-symptomatic and -asymptomatic plants, we have performed a high-throughput sequencing analysis of the transcriptomes and sRNAs from CSD-symptomatic and -asymptomatic citrus plants, all grown in a CSD-affected region. Our work was able to effectively identify both CSDaV and CTV in multiple virus infections and to differentiate two CTV and CSDaV genotypes.

## 2. Materials and Methods

### 2.1. Plant Material

The citrus plants used in this study were monitored since 2003 in CSD-affected groves located in the municipality of Comendador Gomes (southwestern Minas Gerais State), Brazil. Plant tissues from the most representative plants were collected at two different time points, in 2007 and 2014, to construct the transcriptome (RNA-seq) and small RNA libraries, respectively. All plant material was obtained from plants of sweet orange cultivar “Valencia” grafted on different rootstocks that were either susceptible or tolerant to CSD. Fifteen plants were sampled: six trees showed clear CSD symptoms (i.e., occurrence of yellow stain in the rootstock bark) and nine trees were asymptomatic. Genotypes and symptom information are summarized in Table 1. Collected samples were frozen in liquid nitrogen and stored at −80 °C prior to analysis.

### 2.2. RNA Extraction and Sequencing

To construct the RNA-seq libraries, total RNA was extracted using the RNeasy Plant Mini kit (Qiagen, Valencia, CA, USA), according to the manufacturer’s instructions. To construct the small RNA libraries, a high-quality total RNA was obtained by using an adapted CTAB (cetyl trimethylammonium bromide) extraction protocol [15], where the LiCl was replaced by isopropanol (1 vol) in the precipitation phase. The quantity and quality of total RNAs were estimated using a Nanodrop ND-1000 (Thermo Scientific, Wilmington, DE, USA) and 1% agarose gel electrophoresis, respectively. Deep sequencing of both libraries was performed on the Illumina HiSeq 2000 platform (Macrogen, Inc., Seoul, Korea).

### 2.3. RNA-Seq and Small RNA Bioinformatics Analysis

Bioinformatics analyses of each RNA-seq and sRNA data were performed on the CLC Genomic Workbench software package (CLC Bio-Qiagen, Aarhus, Denmark). Trimming of the sRNA data set was done first by removing the adapter sequences. The low-quality reads (limit of 0.05) and the reads shorter than 15 nucleotides (nt) were discarded from the all libraries. Reads were de novo assembled using the CLC Assembly Cell (CLC Bio-Qiagen) and Trinity 2.1.1 [16]. Parameters for optimal assembly were selected based on number and length of the contigs (contiguous sequences) obtained. For both sRNA and RNA-seq libraries, the following settings were used: minimum contig length (100), mismatch cost (2), insertion cost (3), deletion cost (3), length fraction (1), and similarity fraction (0.8). We used word size/k-mer values ranging between 15 and 19 for sRNA and 45 and 65 for RNA-seq. Generated contigs were mapped to the available *C. sinensis* genome (BioProject Accession no. PRJNA225998, URL: https://www.ncbi.nlm.nih.gov/bioproject/PRJNA225998/) to remove contigs related to the host and the unmapped contigs were compared against the non-redundant viral protein database available in the National Center for Biotechnology Information (NCBI) database [17] using Basic Local Alignment Search Tool (BLASTx; default parameters and expected value of 10^−5^ were used) [14]. Potential viral sequences were checked one by one to confirm the BLAST results and all contigs were classified according to the size and sequence with the highest bit score. Contigs that shared high identity with the same virus species were compared against nucleotide database available in NCBI [17] using the BLASTn algorithm to identify the respective potential virus isolates. Based on the largest assembled contigs, number of reads and amino acid identity, predominant viral sequences were screened and selected as candidates for validation.

General genome coverages using merged RNA-seq and sRNA libraries were estimated by mapping the reads against the consensus sequences and viral contigs of the predominant viruses obtained in this study by using the CLC mapping tool (CLC Bio-Qiagen). Open reading frames (ORFs) were predicted using the ORF finder function of the SnapGene software version 3.3 [18]. Comparative analysis between CSD-symptomatic and -asymptomatic plants was also done by merging reads from seven libraries constructed from asymptomatic plants (C1-960, C4-964, C1-963, SN453, SN470, SN476 and SN488) and seven libraries constructed from symptomatic plants (C1-961, C4-965, C1-962, SN464, SN456, SN459, SN479; Table 1), followed by mapping of these two combined libraries to the candidate viral sequences obtained in this study.

### 2.4. Validation of Candidate Viruses

To confirm the presence of the viral sequences identified in the RNA-seq and sRNA libraries, primers designed based on de novo-assembled contigs that showed similarities to viral sequences were used for reverse transcription polymerase chain reaction (RT-PCR) assays. The sequences of all designed primers are shown in Table S1. RNAs extracted from selected CSD-symptomatic and -asymptomatic citrus plants were used as templates and PCR products were analyzed on 0.8% agarose gel and sequenced by Sanger sequencing.

### 2.5. Phylogenetic Analysis

Phylogenetic analysis was performed using amino acid sequences of the RNA-dependent RNA polymerase (RdRP) and Helicase (He) protein, in the case of viral sequences which did not have a conserved domain for RdRP. Each candidate virus was used to compare phylogenetic relationships with other members of the respective viral family, which showed the highest bit score in the BLAST searches. Multiple alignments of amino acid sequences were made by using Clustal X program with the default settings [19]. Phylogenetic trees were constructed using the neighbor-joining (NJ) method in MEGA (version 6.0) [20] with 1000 bootstraps. GenBank accession numbers of the reference sequences used in the phylogenetic analysis are shown in Table S2.

## 3. Results

### 3.1. General Analysis of the RNA-Seq and Small RNA Libraries

From the RNA-seq data, approximately 30 to 37.8 million paired-end reads of 100 base pair (bp) in length were obtained from each library after removing the low-quality reads, yielding assembled viral contigs that varied between 100 and 6109 nt in length (Table 2 and Table 3). Although the RNA-seq analysis showed that the majority of reads were derived from CTV and CSDaV, these libraries have suggested the presence of viral sequences from other several distinct taxa as well. Considering all libraries, we were able to find viral sequences similar to 25 different virus species, representatives of 20 distinct virus families (Table S3). However, several detected virus species (15 out of 25) were represented by only a single short assembled sequence (<300 nt), which were excluded from further analysis. Of the 10 remaining virus species, six showed less than 50% amino acid identity to their homologs in the viral database, suggesting that they might represent novel viral sequences (Table 3). Table S3 provides a list of all viruses from the viral database that showed hits in BLASTx analysis with the assembled sequences obtained from this work. High-throughput sequencing of the sRNA libraries generated approximately 6.8 to 14.2 million usable reads per library after trimming, with a length ranging of 16 to 30 nt. The majority of the assembled viral contigs from these libraries (>90%) was short in length (≤200 bp; Table 2) and the BLASTx searches showed the presence of only CTV and CSDaV as viral sequences in different citrus plants accessed in this study.

### 3.2. Contigs Derived from Citrus Tristeza Virus

**Figure 1:**
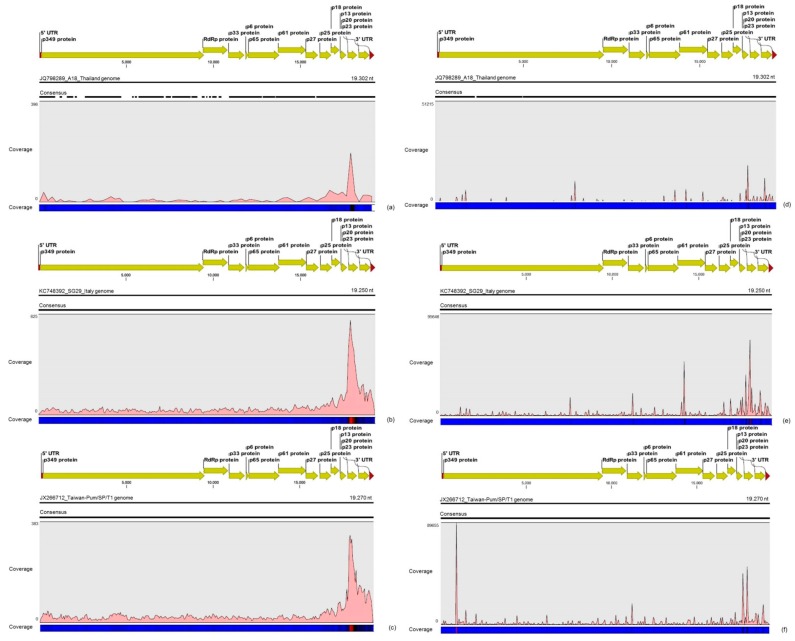
Profile distribution of reads from the RNA-seq (**a**–**c**) and small RNA (sRNA) (**c**–**e**) libraries along the three different *Citrus tristeza virus* (CTV) isolates: A18 (**a**,**d**); SG29 (**b**,**e**); and Taiwan-Pum/SP/1 (**c**,**f**). Genome organization of the CTV references is shown above the respective graphic. Color scale varies from 0 (light blue color) to 100% (red color) of coverage.

CTV was represented by 560 contigs from the RNA-seq libraries that varied between 100 and 3180 nt in length, and by 3996 contigs from the sRNA libraries, with a length ranging of 50 to 539 nt, representing the largest count for any other virus detected in the citrus plants accessed here. Based on the BLASTx and BLASTn searches and number and size of the contigs, we identified predominant assembled sequences that showed high identity (>95%) to three different CTV isolates previously identified as A18 (GenBank accession No. JQ798289), SG29 (GenBank accession No. KC748392) and Taiwan-Pum/SP/T1 (GenBank accession No. JX266712). The mapping of reads from the RNA-seq and sRNA libraries along these three corresponding CTV genomes showed a total of 19,121 and 4,492,130 reads aligned to the reference sequences, respectively (Table 4). Compared to the SG29 and Taiwan-Pum/SP/T1 CTV isolates, a lower distribution of reads was noticed on the A18 CTV isolate genome with higher read counts in areas with high sequence identity among the three different CTV isolates (Figure 1). Therefore, only the SG29 and Taiwan-Pum/SP/T1 CTV isolates from the RB (Resistence Breakdown) [21] and VT lineages [22], respectively, were considered in the further analyses. The read counts mapped on each CTV isolate and the average coverage of the respective genomes are shown in Table 4.

A greater average coverage of the CTV genomes was obtained using reads from sRNA libraries compared to the RNA-seq libraries (Table 4). The density of CTV reads from both RNA-seq and sRNA libraries along the two assumed most predominant CTV genomes (SG29 and Taiwan-Pum/SP/T1) revealed an asymmetric distribution, with a preferential distribution at the 3′ terminal region (Figure 1). CTV reads from RNA-seq libraries gradually increased from the *p25* gene toward *p20*, where we were able to detect a hotspot, and then declined at the *p23* gene and 3′-UTR. Reads from sRNA libraries showed hotspots along the *p13* and *p20* for both CTV isolates, but the frequency and distribution of sRNAs over both references was not identical. For SG29 CTV isolate, hotspots were found at the *p61*, *p33* genes and at the 3′ end of the replicase, whereas for Taiwan-Pum/SP/T1 isolate, we detected hotspots at the *p33* gene and at the beginning of the replicase polyprotein. The CTV consensus sequences were reconstructed under names CTV_SPBR_01 and CTV_SPBR_02 using Taiwan-Pum/SP/T1 and SG29 as reference guide genomes, respectively. The nucleotide sequences of the CTV isolates from this study were deposited in the GenBank database under accession numbers KY110737 and KY110738.

### 3.3. Contigs Derived from Citrus Sudden Death-Associated Virus

CSDaV was represented by 20 contigs from the RNA-seq libraries that varied between 100 and 6109 nt in length, and by 41 contigs from sRNA libraries, all of them with less than 400 nt in length. The largest CSDaV assembled contigs, obtained from the RNA-seq libraries, showed different BLASTn results. Two CSDaV assembled contigs of about 5756 nt and 6109 nt in length showed high identity (>97%) to one of the CSDaV isolates (P15) under accession number DQ185573 in the GenBank, whereas another CSDaV contig (5265 nt) showed higher identity (92%) to the CSDaV isolate available in the GenBank under accession number AY884005. In the re-assembly analysis, a total of 81,700 and 5667 reads from the RNA-seq and sRNA libraries, respectively, were aligned to these two CSDaV reference genomes (Table 4). Different from the results obtained for the CTV sequences, we had some difficulties in obtaining full-genome coverage of the CSDaV genome by assembling the sRNA libraries. The majority of the sRNA libraries (10 out of 13) did not show any CSDaV assembled contigs and a greater average coverage of the CSDaV genomes was obtained using reads only from the RNA-seq libraries (Table 4).

**Figure 2:**
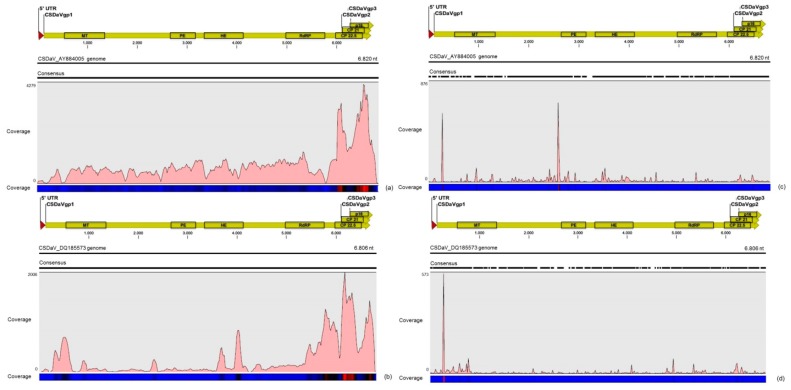
Profile distribution of reads from the RNA-seq (**a**,**b**) and sRNA (**c**,**d**) libraries along the two *Citrus sudden death-associated virus* (CSDaV) isolates under accession numbers: AY884005 (**a**,**c**); and DQ185573 (**b**,**d**). Genome organization of the CSDaV references is shown above the respective graphic. Color scale varies from 0 (light blue color) to 100% (red color) of coverage.

The density of reads from the RNA-seq libraries along the genome of both CSDaV isolates showed a preferential reads distribution at the 3′ terminal region of the CSDaV polyprotein, where the prevalence of the reads was found over the CP domains (Figure 2). Examination of the sRNA profiles revealed a hotspot at the beginning of the CSDaV polyprotein in the 5′ terminal region for both CSDaV isolates and also revealed a notable hotspot in a region close to the beginning of the peptidase domain only for AY884005 CSDaV isolate (Figure 2). The CSDaV consensus sequences obtained were extracted and deposited in the GenBank database under names CSDaV_SPBR_01 and CSDaV_SPBR_02 and accession numbers KY110735 and KY110736, respectively.

### 3.4. Description of the Distinct Viral Sequences Detected in the RNA-Seq Libraries

**Figure 3:**
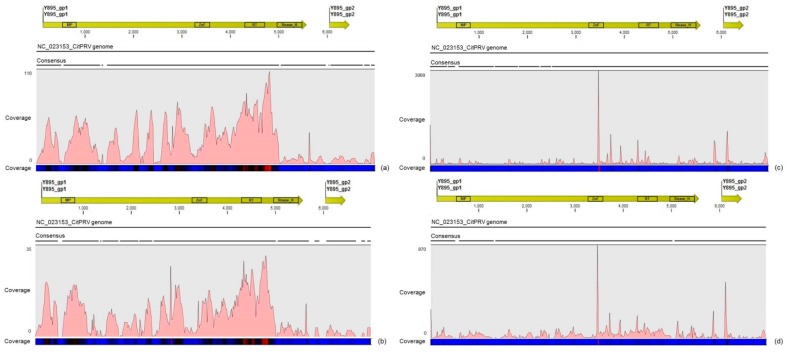
Profile distribution of total reads from the RNA-seq (**a**) and sRNA (**c**) libraries; and reads from combined asymptomatic (**b**) and symptomatic (**d**) libraries along the Citrus endogenous pararetrovirus (CitPRV) genome. Genome organization of the CitPRV reference is shown above the graphics. Color scale varies from 0 (light blue color) to 100% (red color) of coverage.

The other assembled viral contigs (>300 nt) showed to share between 27% and 100% amino acid identity to representatives of the families *Caulimoviridae*, *Dicistroviridae*, *Virgaviridae*, *Flexividae*, *Circoviridae*, *Flaviviridae* and also to unclassified viruses. A few number of short contigs (<400 nt) showed high amino acid identity to Aphid lethal paralysis virus (ALPV; Accession No. NC_004365) and Sclerotinia sclerotiorum deltaflexivirus 1 (SsDFV1; Accession No. KT581451) viruses from the families *Dicistroviridae* and *Flexiviridae*, respectively. However, the re-assembly analyses using these virus genomes as references showed low average coverages in both RNA-seq and sRNA libraries, suggesting a low titre of these viruses in the plants studied here (Table 5). Eight assembled contigs with a length ranging between 339 and 3339 nt showed 67 to 95% amino acid identity to Citrus endogenous pararetrovirus (CitPRV), a virus from the family *Caulimoviridae*. Re-assembly analysis by using the genome of CitPRV as reference sequence (Accession No. NC_023153) resulted in a good average coverage either for RNA-seq (≈29x) and sRNA (≈68x) libraries. Reads from the RNA-seq libraries showed an asymmetric distribution with accumulation of reads over the polyprotein, where the hotspot was found among the reverse transcriptase (RT) and RNase_H conserved domains (Figure 3a). Reads from the sRNA libraries showed a better coverage along the full CitPRV genome with several hotspots, where the highest one was found over the region of the zinc finger (ZnF) conserved domain (Figure 3c). The CitPRV consensus sequence were extracted and deposited in the GenBank database under names CitPRV_SPBR_01 and accession number KY609920.

BLASTx results for the other assembled viral sequences showed a low percentage (22–60%) of amino acid identity to the known virus proteins available in the GenBank, suggesting that these assembled contigs might represent novel viral sequences. Six assembled contigs identified as CtgMarna-1, CtgCirco-1, CtgFlavi-1, CtgUnclass-1, CtgVirga-1 and CtgVirga-2 were used as reference sequences in further mapping analysis (Table 3, Tables S1 and S3). Overall, a low number of reads (less than 400) from the sRNA libraries were found mapping on the viral sequences used as references, which resulted in a low average coverage as well (<1×; Table 5). On the other hand, mapping analysis using reads from the RNA-seq libraries resulted in a better coverage along the viral sequences used as references, ranging between 6× and 114× approximately (Table 5).

### 3.5. Validation of Viral Sequences by RT-PCR and Sanger Sequencing

**Figure 4:**
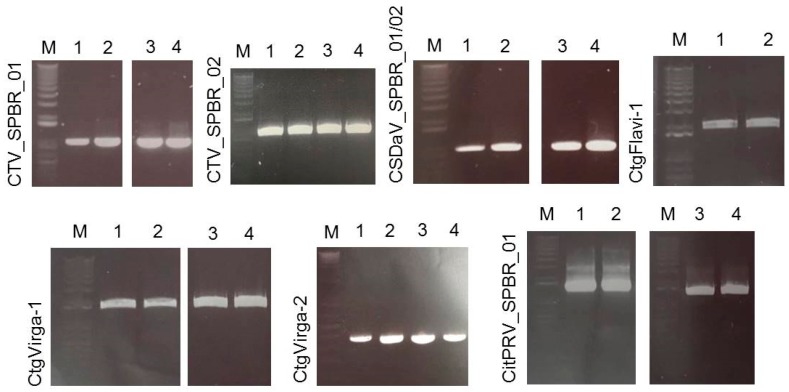
Electrophoretic analysis of virus-specific PCR products amplified from total RNAs extracted from citrus collected in a CSD-affected region. The expected size of the amplified PCR products are: 1001 nucleotides (nt; CTV_SPBR_01); 1095 nt (CTV_SPBR_02); 974 nt (CSDaV); 1929 nt (CtgFlavi-1); 1936 nt (CtgVirga-1); 384 nt (CtgVirga-2) and 1363 nt (CitPRV). **1** and **2**, RNAs from CSD-asymptomatic plants; **3** and **4**, RNAs from CSD-symptomatic plants; **M**, marker 1 kb plus DNA ladder.

RNA samples extracted from four different citrus plants (two CSD-symptomatic and two asymptomatic) were used to confirm the presence of the viruses detected in the sRNA and RNA-seq libraries by RT-PCR and Sanger sequencing. Based on the assembled viral contigs that we obtained, specific primers were designed (Table S1) to differentiate the two dominant CTV genotypes; to detect the CSDaV, CitPRV, ALPV and SsDFV1 viruses; and to confirm the presence of the viral contigs identified as CtgMarna-1, CtgCirco-1, CtgFlavi-1, CtgUnclass-1, CtgVirga-1 and CtgVirga-2. Positive RT-PCR results were obtained for both CTV genotypes (CTV_SPBR_01 and 02), CSDaV, CitPRV, CtgVirga-1 and CtgVirga-2 in RNA samples from both symptomatic and asymptomatic plants (Figure 4). The presence of the contig identified as CtgFlavi-1 was validated only in RNAs from asymptomatic plants (Figure 4). The RT-PCRs to detect the presence of the ALPV, SsDFV1, CtgMarna-1, CtgCirco-1 and CtgUnclass-1 viral sequences were negative for all tested plants.

### 3.6. Sequence and Phylogenetic Analysis of the Viral Sequences Related to the CTV, CSDaV and CitPRV, the Known Viruses Detected in This Study

**Figure 5:**
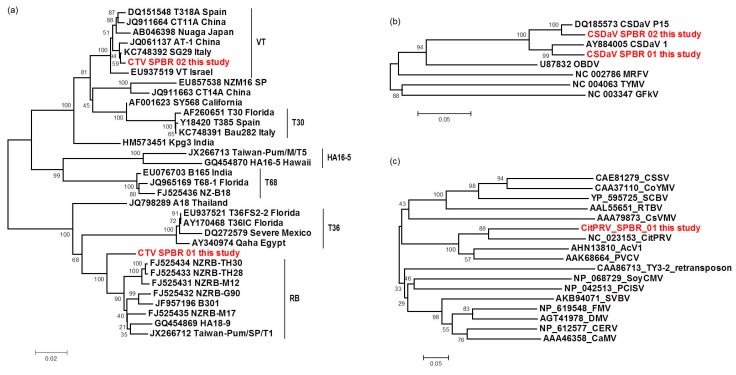
Phylogenetic relationships among RNA-dependent RNA polymerase (RdRP) (**a**,**b**) and reverse transcriptase (**c**) amino acid sequences from representative isolates of: CTV (**a**); CSDaV (**b**); and CitPRV (**c**), including the respective viral sequences identified in this study. Bootstrap values are shown as percentages and the viral sequences obtained in this study are highlighted in red.

The complete CTV consensus sequences obtained in this study was found to be structurally identical to known CTV isolates, both with 12 ORFs. The CTV_SPBR_01 genotype showed to share 99% identity to TaiwanPum/SP/1 isolate and was found to be 19,251 nt in length, including 104 nt in the 5′-UTR and 258 in the 3′-UTR. The CTV_SPBR_02 complete consensus sequence showed 99% sequence identity to the SG29 isolate and was found to be 19,243 nt in length, including 102 nt in the 5′-UTR and 273 in the 3′-UTR. Phylogenetic analysis based on the RdRP amino acid sequences of the 31 selected previously published CTV genome sequences (Table S2) and the two genotypes sequenced in this study, placed CTV_SPBR_01 closer to the isolates from the RB lineage (previously reported for its ability to break down Poncirus trifoliata resistance [21]), which includes NZRB isolates, an isolate from Hawaii (HA18-9), Taiwan (TaiwanPum/SP/1) and Puerto Rico (B301); whereas CTV_SPBR_02 was found to cluster within the VT lineage, which includes the severe isolates from Spain (T318A), Asian (AT-1, CT11A and Nuaga), Israel (VT) and Italy (SG29) (Figure 5a).

The complete consensus sequences of the two CSDaV genotypes obtained in this study showed similar structure to the previously reported CSDaV genome sequences, both showing a large ORF encoding for a polyprotein and a small ORF representing a putative movement protein, (p16). The CSDaV_SPBR_01 genotype showed to share 93% identity to AY884005 CSDaV isolate and was found to be 6802 nt in length, including 108 nt in the 5′-UTR and 127 in the 3′-UTR, excluding the poly(A) tail. The CSDaV_SPBR_02 complete consensus sequence showed 97% sequence identity to the DQ185573 CSDaV isolate and was found to be 6803 nt in length, including 109 nt in the 5′-UTR and 127 in the 3′-UTR, excluding the poly(A) tail. A phylogenetic tree was constructed based on RdRP amino acid sequences from the two previously reported CSDaV genome sequences, the two CSDaV sequences from this study and from the other four members of the family *Tymoviridae* (Table S2). This placed the CSDaV_SPBR_01 and CSDaV_SPBR_02 in different clades, closer to AY884005 and DQ185573 CSDaV isolates, respectively (Figure 5b). RT amino acid sequence was obtained from the CitPRV_SPBR_01 consensus sequence and included in a comparative phylogenetic analysis with other members of the family *Caulimoviridae* and Ty3 retrotransposon from *Saccharomyces cerevisiae* to confirm the high phylogenetic relatedness to the respective endogenous pararetrovirus, which was clustered in the same clade with high supporting bootstrap value (90%) (Figure 5c).

### 3.7. Phylogenetic Analysis and Preliminary Genome Characterization of the Unknown Viral Sequences Identified in This Study

**Figure 6:**
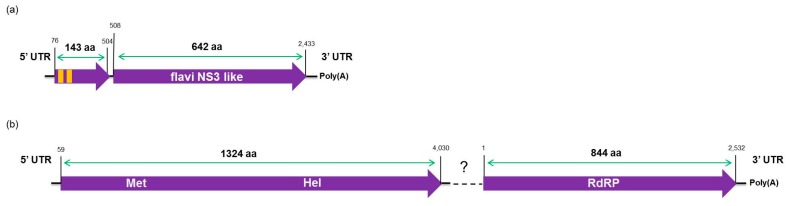
Schematic illustration of the predicted partial genome organization of the two putative novel viruses identified in this study: (**a**) a putative segment 1 of the Citrus jingmen-like virus (CJLV) genome showing two predicted open reading frames (ORFs; purple arrows) and two predicted transmembrane regions (orange boxes); and (**b**) partial Citrus virga-like virus (CVLV) predicted genome showing two predicted ORFs (purple arrows). The detected conserved domains and the amino acid length of each ORF are indicated.

**Figure 7:**
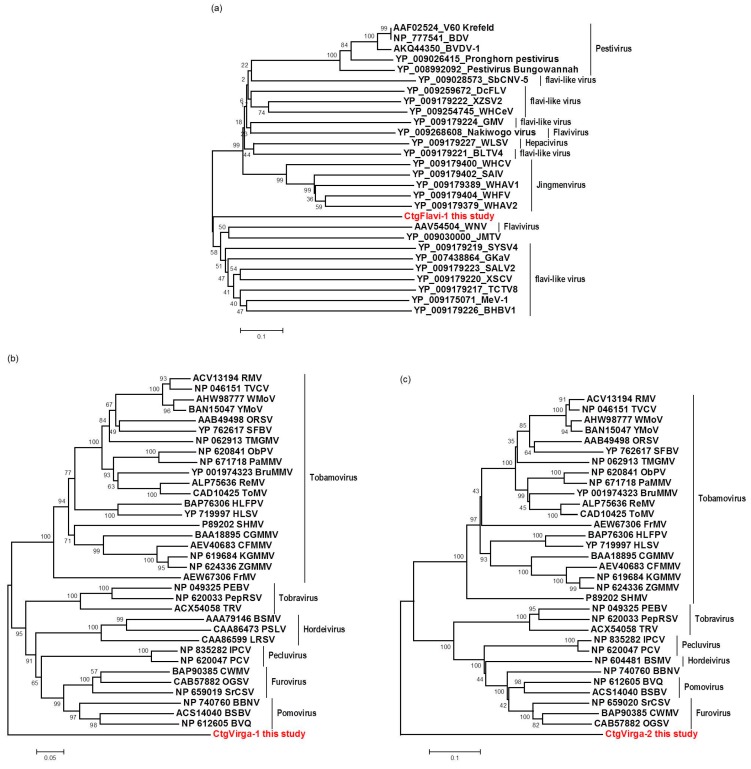
Phylogenetic relationships among helicase (**a**,**b**) and RdRP (**c**) amino acid sequences from representative isolates of the families *Flaviviridae* (**a**) and *Virgaviridae* (**b**,**c**), including the respective viral sequences identified in this study. Bootstrap values are shown as percentages and the viral sequences obtained in this study are highlighted in red.

The viral contigs identified as CtgFlavi-1, CtgVirga-1 and CtgVirga-2 were assembled by the bioinformatics analysis of RNA-seq data and subsequently confirmed by RT-PCR and Sanger sequencing in the RNA samples. The contig CtgFlavi-1 was found to be 2512 nt in length, including the 75 nt in the 5′-UTR and 71 nt in the 3′-UTR, excluding the poly(A) tail. Two ORFs were predicted: ORF1 (position 76 to 504) and ORF2 (position 508 to 1926). BLASTx analysis did not detect any putative conserved domains for either ORFs, but ORF2 showed a low sequence identity (27%) to a nonstructural protein NS3 of the Nakiwogo virus, an insect-specific flavivirus from the family *Flaviviridae* [23]. However, the CtgFlavi-1 viral sequence showed several similar characteristics to the segment 3 of the Jingmen tick virus (JMTV), a segmented tick-borne virus, also from the family *Flaviviridae* [24]. Examples of these similar characteristics are: the partial genome sequences resembling flavivirus nonstructural NS3 protein, the protein size at about 800 amino acids, the presence of UTRs and poly(A) tail, and the presence of two transmembrane regions, predicted by the TMHMM program 2.0 [25] (Figure 6a). Phylogenetic analysis based on the nonstructural protein NS3 or helicase protein of the 27 selected genome sequences from the different members of the family *Flaviviridae* (Table S2), placed CtgFlavi-1 in a separate clade between Jingmenviruses and Flaviviruses, closer to the West Nile virus and to the Jingmen tick virus (Figure 7). This phylogenetic distance and the low protein identity obtained from the BLASTx analysis suggest that the viral sequence CtgFlavi-1 might be a genome segment belonged to a novel virus from the family *Flaviviridae*. However, all attempts to find other fragments that could be associated to CtgFlavi-1 sequence failed.

The CtgFlavi-1 sequence was deposited in the GenBank as a segment 1 belonging to a putative segmented novel virus tentatively named Citrus jingmen-like virus (CJLV; Accession number KY110739). The CtgVirga-1 contig seems to be almost completed with 4097 nt in length, including 58 nt in the 3′-UTR and 67 nt in the 5′-UTR, and has shown two putative conserved domains in the BLASTx analysis, encoding for methyltransferase and helicase proteins. The CtgVirga-2 contig showed a putative conserved domain encoding for RdRP protein and was found to be 2626 nt in length, including 86 nt in the 3′-UTR, excluding the identified poly(A) tail, but the 5′ terminal region showed to be not fully completed because the sequence in this region still in ORF. Based on the genome organization of members from the family *Virgaviridae*, a positive-sense single strand RNA plant virus family, we have assumed that both CtgVirga-1 and CtgVirga-2 contigs might be part of the same virus, probably from the RNA1 of a segmented genome. However, attempts to join the two contigs have not shown a conclusive result and attempts to find sequences related to *Virgaviridae* movement and coat protein in the RNA-seq data have failed as well. Comparative amino acid sequences analysis based on the helicase and the RdRP amino acid sequences of the selected genomes (34 for the helicase and 32 for the RdRP) from the different members of the family *Virgaviridae* (Table S2) was done including the CtgVirga-1 and CtgVirga-2 contigs, respectively. Both helicase and RdRP phylogenetic trees placed the CtgVirga-1 and CtgVirga-2 contigs in a separated clade, phylogenetically distant to the other genus of the family *Virgaviridae*, suggesting that these contigs might be part of a novel virus that might represent a novel genus within the family *Virgaviridae*. The CtgVirga-1 and CtgVirga-2 sequences were deposited in the GenBank as un-joined fragments belonged to a putative novel virus tentatively named Citrus virga-like virus (CVLV) (Accession numbers KY110740 and KY110741, respectively). Schematic genome organization and predicted ORFs are shown in Figure 6.

### 3.8. Comparison of Viral Sequences Derived from CSD-Symptomatic and Asymptomatic Plants

**Figure 8:**
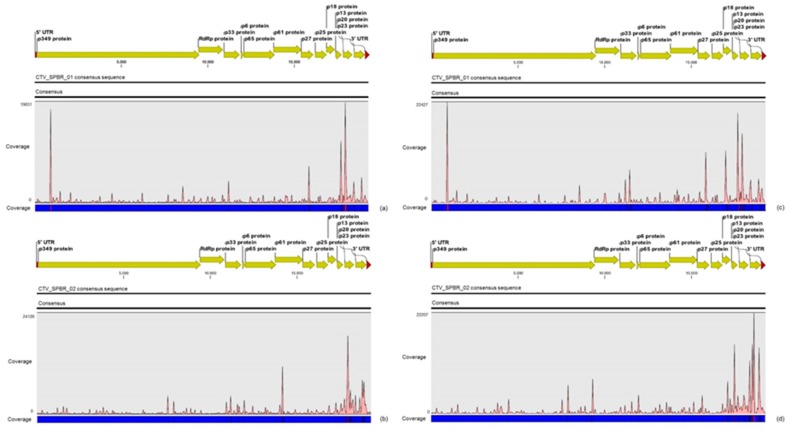
Profile distribution of reads from combined asymptomatic (**a**,**b**) and symptomatic (**c**,**d**) libraries along the two CTV consensus sequence obtained in this study: CTV_SPBR_01 (**a**,**b**); and CTV_SPBR_02 (**b**,**d**). Genome organization of the CTV references is shown above the respective graphic. Color scale varies from 0 (light blue color) to 100% of coverage (red color).

The consensus sequences identified as CTV_SPBR_01, CTV_SPBR_02, CSDaV_SPBR_01, CSDaV_SPBR_02 and CitPRV_SPBR_01, as well as the contigs identified as CtgFlavi-1, CtgVirga-1 and CtgVirga-2 were used as references in comparative mapping analysis between libraries constructed from CSD-symptomatic and -asymptomatic plants. For both CTV consensus sequences used as references, the average coverages were found to be at about 1.2 times higher in libraries constructed from symptomatic plants (Table 6). Although this difference is not likely to be significant, we did see an asymmetric read distribution along the both CTV consensus sequences when libraries from symptomatic and asymptomatic plants were compared. From the 5′ terminal to the *p25* region, the read distribution along the CTV_SPBR_01 consensus sequence was similar for both asymptomatic and symptomatic libraries, where hotspots were found over the 5′ terminal region of the ORF1a and over the *p27* region. In the 3′ terminal region, an accumulation of reads from asymptomatic libraries was found in the *p13* and *p20* region, which *p20* showed to have the highest coverage, whereas the hotspots for symptomatic libraries were found to be in the *p18*, *p13* and *p20*, which the highest coverage was over the *p13* region. The mapping on the CTV_SPBR_02 consensus sequence showed that the hotspots for asymptomatic libraries were found over the *p61* and *p20* region, whereas notable hotspots were detected over the *p13* and *p23* for the symptomatic libraries (Figure 8).

**Figure 9:**
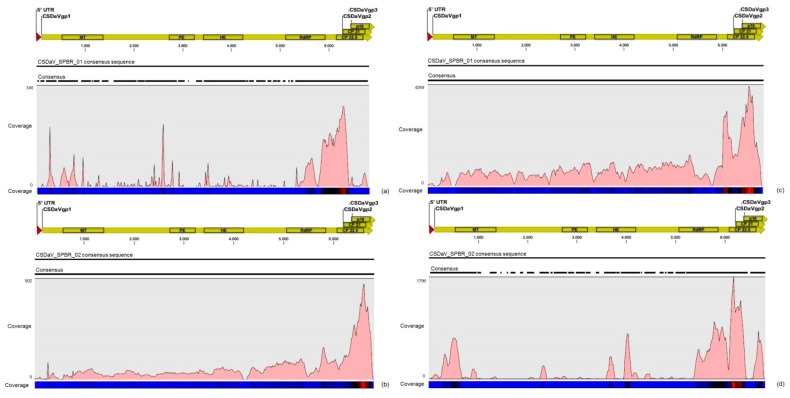
Profile distribution of reads from combined asymptomatic (**a**,**b**) and symptomatic (**c**,**d**) libraries along the two CSDaV consensus sequence obtained in this study: CSDaV_SPBR_01 (**a**,**b**); and CSDaV_SPBR_02 (**b**,**d**). Genome organization of the CSDaV references is shown above the respective graphic. Color scale varies from 0 (light blue color) to 100% (red color) of coverage.

Different from the results obtained for CTV, the mapping on the CSDaV_SPBR_01 and CSDaV_SPBR_02 revealed great differences on average coverage and read distribution between them, and also between the libraries from symptomatic and asymptomatic plants. The average coverage of the CSDaV_SPBR_01 sequence using reads from the symptomatic libraries showed to be at about 29 times higher than the average coverage estimated with mapped reads from the asymptomatic libraries (Table 6). Accumulation of reads from asymptomatic libraries was found in several points along the CSDaV_SPBR_01 sequence, but the coverage even for these regions was low (Figure 9). Distribution of the reads from symptomatic libraries showed to be more abundant in the 3′ terminal region of the CSDaV sequence, where hotspots were detected over the coat proteins and *p16* region. The average coverage of the CSDaV_SPBR-02 consensus sequence was only about 1.6 times higher in symptomatic libraries, compared to the asymptomatic libraries. The mapping of reads from asymptomatic libraries showed a symmetric distribution of the reads along the CSDaV_SPBR_02 sequence until coming to the region encoding the CP and p16 proteins, where we detected the presence of a hotspot. On the other hand, mapped reads from symptomatic libraries on the CSDaV_SPBR_02 sequence showed an asymmetric distribution with several points of read accumulation around the 5′ terminal, helicase, RdRP and CP regions.

Analysis of the read distribution profile on the CitPRV_SPBR_01 consensus sequence showed that symptomatic libraries have an average coverage at about ≈5× higher than in asymptomatic libraries. It has also noticed that symptomatic libraries seem to have higher number of small RNAs, whereas asymptomatic libraries showed more mapped reads from the RNA-seq libraries (Figure 3). Re-assembly analysis using mapped reads from symptomatic and asymptomatic libraries on the viral contigs CtgFlavi-1, CtgVirga-1 and CtgVirga-2 showed results opposite to what we obtained for CTV, CSDaV and CitPRV reads. Surprisingly, the average coverage of the CtgFlavi-1 contig showed to be at about 760 times higher using reads from asymptomatic libraries, compared to the symptomatic libraries (Table 6). Similarly, reads mapped on the contigs CtgVirga-1 and CtgVirga-2 showed to be more abundant in the asymptomatic libraries, with an average coverage around 280 and 1040 times higher, respectively, compared with symptomatic libraries (Table 6).

## 4. Discussion

Our study demonstrated a putative association of CSD-symptomatic plants with a specific CSDaV genotype and with Citrus endogenous pararetrovirus (CitPRV) as well. We were also able to identify two putative novel viruses that were shown to be more associated with CSD-asymptomatic plants. This work provides new insights into the role of the identified viruses in citrus plants affected by CSD, which will be able to contribute to further studies.

In this work, Illumina high-throughput sequencing of the transcriptome and sRNAs from citrus plants grown in a region affected by citrus sudden death disease has allowed us to identify and compare viral sequences presenting in these plants. The deep sequencing analyses were sensitive and sufficient to identify the predominant viruses, to obtain information about their genetic diversity, and to demonstrate the presence of putative novel and low-titer viruses. CTV was the most predominant virus identified here, represented by 97.4% of total reads, followed by CSDaV, which corresponded to 1.94% of the reads, CitPRV with 0.53% of the reads, and other viruses represented by 0.13% of the reads. The presence of the two first mentioned viruses was not a surprising finding because in attempts to discover the causal agent of CSD, both of these viruses were detected and associated with CSD-affected plants. Maccheroni et al. [9] reported a high correlation at 99.7% of the assessed plants between CSD symptoms and the presence of CSDaV, but the role of this virus in CSD is not yet clear. CTV is an endemic virus in Brazil, mainly due to the cross-protection program and the presence of aphid transmitters [26]. Previous published works have used different approaches to identify an isolate or new variant of CTV associated with CSD, but all attempts have failed so far [2,6,7,8]. Although previous works have shown that citrus plants affected by CSD are infected by a mixed population of divergent CTV variants [2,7,8,9], it is still unknown which specific CTV genotypes are present in those plants. To our knowledge, this is the first high-throughput sequencing-based study of the viral sequences present in citrus plants affected by the CSD disease. Our work reveals mixed viral infections in both CSD-symptomatic and -asymptomatic plants, including CTV, CSDaV, CitPRV and two putative novel viruses tentatively named in this study as CJLV and CVLV.

For the first time, the full consensus sequences of the two predominant Brazilian CTV genotypes present in those CSD-affected plants were obtained and identified here as CTV_SPBR-01 and CTV_SPBR_02. Phylogenetic analysis clustered CTV_SPBR_01 within RB-like CTV isolates, whereas CTV_SPBR_02 was clustered within VT-like CTV isolates. Both of the resistance breaking (RB) and VT strains have been characterized as severe or aggressive strains, which are associated with decline symptoms of citrus trees propagated on sour orange rootstock (*Citrus aurantium* L.) or stem pitting (SP) of the scion regardless of the rootstocks [27,28,29,30]. Although re-assembly analysis comparing CTV mapped reads between libraries from asymptomatic and symptomatic plants did not show significant differences regarding average coverage values, differences on the read distribution and hotspot regions between these two libraries were noticed. Similar to the results obtained here, previous works reported that CTV infection induces accumulation of sRNAs mapping preferentially at the 3′-terminal region of the viral genome [21,31]. Interestingly, mapping reads from asymptomatic libraries for both CTV genotypes identified here has shown a hotspot over the silencing suppressor gene p20, besides other lower hotspots as well, whereas mapping reads from symptomatic libraries showed an increased read coverage over the host range associated genes: the p13, p18 and p33 when CTV_SPBR_01 consensus sequence was used as reference, and the *p13*, when CTV_SPBR_02 was used as reference. CTV_SPBR_02 also showed a hotspot over the p23 gene, which is a multifunctional gene and is also associated with silencing suppressor activity [32]. Based on these results, the association of these two predominant, severe-like CTV isolates with CSD-symptomatic plants is not clear, however, the results led us to think about a new question concerning these CTV isolates: Could these isolates be the helpers in mixed virus infections by using their silencing suppressor and host range genes/proteins to facilitate the systemic infection of the other virus(es)? CSDaV could be this other virus and involved with CSD. Interestingly, the CSDaV consensus sequence obtained from libraries constructed from the symptomatic plants (CSDaV_SPBR_01) showed to be phylogenetically distant from the CSDaV consensus sequence extracted from the asymptomatic libraries (CSDaV_SPBR_02), showing at about 13% nucleotide diversity between them, which is consistent with our previous work on the CSDaV genetic diversity [33]. Furthermore, a remarkable 29 times higher average coverage was found in mapping reads from symptomatic libraries on the CSDaV_SPBR_01 consensus sequence, compared to mapping reads from asymptomatic libraries. The average coverage of the CSDaV_SPBR_02 genotype using reads from the symptomatic plants was only 1.6 times higher than mapping reads from asymptomatic libraries on the same genotype. These results strongly support an association of CSDaV with CSD symptoms and suggest that there is a specific CSDaV genotype that could be more associated with this disease.

Another interesting result came from the comparative analysis between mapped reads from the asymptomatic and symptomatic libraries on the endogenous CitPRV genome. Besides the higher average coverage of this virus in symptomatic libraries (at about five times), it was also noticed that symptomatic libraries have higher number of small RNAs, compared to the asymptomatic libraries. It has been shown that other plant pararetroviruses, such as endogenous Petunia vein clearing virus (PVCV) and Tobacco vein clearing virus (TVCV), can be in some way induced, culminating to the development of viral symptoms and sRNA accumulation [34,35,36]. Although the difference regarding the average coverage of the CitPRV between symptomatic and asymptomatic libraries was lower than that we obtained for CSDaV, this result cannot be ignored. As far as we know, this is the first time that CitPRV was identified in citrus plants in Brazil, and it represents the initial step in studying the possible role of CitPRV in CSD symptoms in these plants.

The high-throughput sequencing approach also allowed the identification of two putative novel viruses infecting the plants studied here, which shows low amino acid identity to viruses from the families *Flaviviridae* and *Virgaviridae*. Interestingly, although the genomes of both viruses are not completed using our data here, results obtained from the re-assembly analysis on the contigs from the CJLV and CVLV demonstrated a remarkable higher average coverage for both viruses in asymptomatic libraries. Besides that, it was also observed a higher diversity of viral sequences in these libraries. Of 27 viral species identified in the BLASTx analysis using assembled contigs obtained in this study as queries, 21 of them were found only in asymptomatic libraries. The lower viral diversity in libraries constructed from symptomatic plants is might be attributed to a strong competition among different viruses within the host for adequate replication conditions. Our results might suggest two things: (1) in the CSD-affected plants, viruses that are associated to developing CSD symptoms (i.e., CTV, CSDaV and/or CitPRV) are the fittest viruses, eliminating or suppressing other viruses from the within-host competition; and (2) in plants not affected by CSD, other viruses (i.e., CJLV and/or CVLV) could play a role in suppressing infections by virus(es) putatively associated in developing CSD symptoms.

In summary, this work has shown that high throughput sequencing analysis, combining data from RNA-seq and sRNA libraries, provided a wide range of information and it was a valid approach to identify and compare viral sequences in citrus plants grown in regions affected by CSD. The correlation of the viruses with the CSD disease indicated a higher association of the CSD-symptomatic plants with a specific CSDaV isolate/genotype and a likely association with CitPRV. We have identified two putative novel viruses that, interestingly, showed to be more associated with the CSD-asymptomatic plants. This study also contributed to describing, for the first time, the specific predominant CTV isolates/genotypes infecting citrus plants grown in the CSD-affected region. All data obtained in this work could together provide new insights into the role of the identified viruses in citrus plants affected by CSD and contribute to further epidemiological studies.

## Figures and Tables

**Table 1:** Citrus plants used to assess the viral sequences by using high-throughput sequencing. All experimental plants had *Citrus sinensis* cv. Valencia scions that were grafted onto different rootstocks. Rootstock varieties, type of collected plant tissue and type of library constructed are shown.

	Canopy/Rootstock	Collected Tissue	Type of Library Constructed	Library ID
Asymptomatic plants	^1^ Valencia/Rough lemon	Roots	sRNA	SN453
	Valencia/Citrandarin Cleopatra × Rubidoux	Leaves	sRNA	SN468
	^1^ Valencia/Rough lemon	Leaves	sRNA	SN470
	Valencia/Trifoliata Tristeno	Leaves	sRNA	SN473
	Valencia/Rangpur lime × Swingle A	Leaves	sRNA	SN476
	Valencia/Sunki mandarin	Leaves	sRNA	SN483
	Valencia/Sunki × Cleopatra	Leaves	sRNA	SN486
	Valencia/Swingle	Leaves	sRNA	SN488
	^2^ Valencia/Rangpur lime	Leaves	RNA-seq	C1-960
	^2^ Valencia/Rangpur lime	Roots	RNA-seq	C4-964
	Valencia/Sunki of China	Leaves	RNA-seq	C1-963
Symptomatic plants	^3^ Valencia/Rough lemon	Roots	sRNA	SN464
	^3^ Valencia/Rough lemon	Leaves	sRNA	SN456
	Valencia/Rangpur lime × Swingle A	Leaves	sRNA	SN459
	Valencia/Citrus pennivesiculata	Leaves	sRNA	SN462
	Valencia/Rangpur lime	Leaves	sRNA	SN479
	^4^ Valencia/Rangpur lime	Leaves	RNA-seq	C1-961
	^4^ Valencia/Rangpur lime	Roots	RNA-seq	C4-965
	Valencia/Sunki of China	Leaves	RNA-seq	C1-962
Total of plants: 15; total of samples: 19				

^1^ Same plant; ^2^ Same plant; ^3^ Same plant; ^4^ Same plant. sRNA: small RNA; RNA-seq: RNA sequencing.

**Table 2:** Reads and contigs count information of each RNA-seq and sRNA library. The number of assembled viral contigs was organized according to their size.

Library ID	No. of Reads after Trimming	No. of Exogenous Reads	^3^ No. of Putative Viral Contigs Detected in an Overall Screening	^4^ No. of Viral Contigs Detected in an Individual Screening		
				≤200 nt	Between 201 to 999 nt	≥1000 nt
^1^ C1-960	37,811,400	3,826,317	40,187	76	41	7
^1^ C1-961	37,380,448	3,613,441	29,483	74	37	9
^1^ C1-962	36,452,005	3,528,784	30,834	72	32	6
^1^ C1-963	29,942,484	3,027,793	38,028	54	25	2
^1^ C4-964	35,511,705	3,741,954	33,719	10	4	4
^1^ C4-965	35,386,080	3,617,222	39,012	24	13	0
^2^ SN453	8,091,654	756,302	776	323	19	0
^2^ SN456	8,949,837	1,117,451	395	263	5	0
^2^ SN459	9,899,316	1,065,849	571	334	6	0
^2^ SN462	6,233,982	836,217	267	198	5	0
^2^ SN464	9,042,291	782,698	821	178	29	0
^2^ SN468	8,843,660	897,286	626	384	11	0
^2^ SN470	6,866,756	1,041,001	410	329	4	0
^2^ SN473	11,892,105	1,597,924	533	311	41	0
^2^ SN476	9,076,165	917,605	631	408	14	0
^2^ SN479	11,649,840	1,232,264	733	163	24	0
^2^ SN483	8,765,391	949,445	471	326	13	0
^2^ SN486	11,001,152	1,127,439	555	242	13	0
^2^ SN488	14,231,970	1,446,481	783	507	20	0
Total	337,028,241	35,123,473	218,835	4276	356	28

^1^ RNA-seq libraries ID; ^2^ sRNA libraries ID; ^3^ Number of contigs that showed Basic Local Alignment Search Tool (BLASTx) hits to any viral proteins in the National Center for Biotechnology Information (NCBI) database in the first screening by using all contigs simultaneously; ^4^ Number of viral contigs that showed BLASTx hits to any viral proteins in NCBI database in the second screening by using contigs individually; nt, nucleotide.

**Table 3:** Contig counts and contig length for each viral species (>300 nt in length) identified in the BLASTx analysis of the total data set.

Closely Related Viruses	Family	No. of Contigs	Contigs Length (nt)	From RNA-Seq Libraries	From sRNA Libraries	Maximum % aa Identity
Citrus tristeza virus	*Closteroviridae*	4556	50–3180	560	3996	92
Citrus sudden death-associated virus	*Tymoviridae*	61	50–6109	20	41	98
Marine RNA virus SF-2	*Marnaviridae*	1	1400	1	0	22
Po-Circo-like virus 51	*Circoviridae*	1	305	1	0	43
Aphid lethal paralysis virus	*Dicistroviridae*	6	115–343	6	0	97
Nakiwogo virus	*Flaviviridae*	1	2512	1	0	27
Sclerotinia sclerotiorum deltaflexivirus 1	*Flexiviridae*	5	101–329	5	0	62
Citrus endogenous pararetrovirus	*Caulimoviridae*	8	339–3339	8	0	72
Fusarium graminearum deltaflexivirus 1	Putative *Deltaflexiviridae*	2	153–262	2	0	71
Boutonnet virus	unclassified viruses	2	423–434	2	0	36
Beet virus Q	*Virgaviridae*	1	4097	1	0	33
Chinese wheat mosaic virus	*Virgaviridae*	1	2626	1	0	28

nt: nucleotides; aa: amino acid.

**Table 4:** Comparison of the re-assembly data for the two dominant virus species identified in this study (Citrus tristeza virus; CTV and Citrus sudden death-associated virus; CSDaV). Read counts from the simultaneous re-assembly analysis are shown for the three assumed predominant CTV isolates and for the two CSDaV isolate.

Virus	Reference Isolate	sRNA Simultaneous Re-Assembly			RNA-Seq Simultaneous Re-Assembly		
		Read Count	Percentage Read Count	Average Coverage	Read Count	Percentage Read Count	Average Coverage
CTV	A18	711,217	15.8%	≈740×	2,450	12.8%	≈13×
	Taiwan-Pum	1,800,699	40.1%	≈1870×	6,789	35.5%	≈35×
	SG29	1,980,214	44.1%	≈2060×	9,882	51.7%	≈50×
	Total	4,492,130	100%	−	19,121	100%	−
CSDaV	AY884005	3944	69.6%	≈12×	59,916	73.3%	≈810×
	DQ185573	1723	30.4%	≈5×	21,784	26.7%	≈295×
	Total	5667	100%	−	81,700	100%	−

**Table 5:** Comparison of re-assembly data among different viral sequences identified in this study. Read count and average coverage from the simultaneous re-assembly analysis are shown for each viral sequence.

^1^ Reference Viral/Contig Sequence	sRNA Simultaneous Re-Assembly		RNA-Seq Simultaneous Re-Assembly	
	Read Count	Average Coverage	Read Count	Average Coverage
ALPV	387	0.52x	113	1.1x
CitPRV	21,693	68.22x	2196	28.88x
SsDFV1	227	0.49x	3	0.02x
CtgCirco-1	12	0.53x	25	6.57x
CtgFlavi-1	89	0.53x	3144	113.95x
CtgMarna-1	68	0.64x	83	5.82x
CtgUnclass-1	103	3.37x	189	41.4x
CtgVirga-1	163	0.59x	2297	51.4x
CtgVirga-2	105	0.57x	1723	61.8x

^1^ Viral sequences (downloaded from the GenBank) or contig sequences (obtained in this study) used as references in re-assembling analysis by mapping reads from the RNA-seq and sRNA libraries.

**Table 6:** Re-assembly data among different viral sequences identified in this study by mapping reads from asymptomatic and symptomatic combined libraries for comparative analysis. Read count and average coverage are shown for each viral sequence.

Reference Viral Sequence	Asymptomatic Libraries Re-Assembly		Symptomatic Libraries Re-Assembly	
	Read Count	Average Coverage	Read Count	Average Coverage
CTV_SPBR_01	418,902	442.32x	525,380	563.19x
CTV_SPBR_02	553,150	584.91x	633,371	693.78x
CSDaV_SPBR_01	3934	26.49x	58,532	767.43x
CSDaV_SPBR_02	8844	109.53x	14,350	182.44x
CtgFlavi-1	3182	114.2x	28	0.15x
CtgVirga-1	2582	56.14x	59	0.2x
CtgVirga-2	1791	62.18x	12	0.06x
CitPRV_SPBR_01	721	8.35x	8325	41.65x

## Supplementary Materials

The following are available online at www.mdpi.com/1999-4915/9/4/92/s1, **Table S1:** Primer sequences designed based on de novo-assembled contigs to validation assays; **Table S2:** Accession numbers of the reference sequences used in the phylogenetic analysis; **Table S3:** Query coverage and maximum amino acid identity obtained from the BLASTx analysis using the assembled viral contigs from this work as query sequences.
