# Supplementary material for: Deep Sequencing Analysis of RNAs from Citrus Plants Grown in a Citrus Sudden Death-Affected Area Reveals Diverse Known and Putative Novel Viruses
Source: Viruses. 2017 Apr 24;9(4):92. doi: 10.3390/v9040092 (PMC5408698; doi:10.3390/v9040092)
Supplement: Supplementary file 1 [file viruses-09-00092-s001.docx]

**Table S1.** Primer sequences designed based on de novo-assembled contigs to validation assays.

| **Reference viruses or viral contigs for primer designing** | **Closely related species/Family** | **^1^ Primer sequences (5'–3')** | **PCR product expected size** | |
| --- | --- | --- | --- | --- |
| **CTV_SPBR_01** | *Citrus tristeza virus/Closteroviridae* | F - CCGAAGAAGTGACACCAGTCTGTAAG  R - AGAAGCCGCACCAGTAACGTACTTAG | | 1001 nt |
| **CTV_SPBR_02** | *Citrus tristeza virus/Closteroviridae* | F - GGACTTGTAGAAGACGGCAAGAAGTC  R - CCGGCGGTTTTAACAACGTGTTTAG | | 995 nt |
| **CSDaV_SPBR_01/02** | *Citrus sudden death-associated virus/Tymoviridae* | F - TCCAACCAGTTCCCACACATGGACAA  R - GCGTACCGACCCCTTTCTTTCTTGAA | | 974 nt |
| **ALPV** | *Aphid lethal paralysis virus/Dicistroviridae* | F - AACAAGACTTGGCCCGTGTATCTGAC  R - CTACAGAGATGCCTTTGTTCATGTGCG | | 320 nt |
| **CitPRV** | *Citrus endogenous pararetrovirus/Caulimoviridae* | F - TGACATCCTGCACCAGATCAAACACC  R - CCATTCGGATGAGAAAATGGTGTCC | | 1363 nt |
| **SsDFV1** | *Sclerotinia sclerotiorum deltaflexivirus 1/Flexiviridae* | F - CCCCAGATGGAAATTCAGCCTGATC  R - TCAACATTGAATGCATGCGCATGCC | | 300 nt |
| **CtgCirco-1** | *Po-Circo-like virus 51/Circoviridae* | F - CGTATGTATCAGGCACTTTGGGAGC  R - GGATATTCATCGGAGCCATAATGGC | | 200 nt |
| **CtgFlavi-1** | *Nakiwogo virus/Flaviviridae* | F - TAATCTGGTCTTTAGCCCTCTCTGGG  R - TATGCCTCCAGTTCCCAGGTGGTACTA | | 1929 nt |
| **CtgMarna-1** | *Marine RNA virus SF-2/Marnaviridae* | F - CGGACGCCATGTCAAATTTGCATTC  R - CCGTTTTCAGCCTTGAAGAGAGGAGA | | 1300 nt |
| **CtgUnclass-1** | *Unclassified virus* | F - ATTCGGTCGCTTCACCACTATCGATG  R - GAAGAAAGGAACCGGGAGCCATGAAA | | 250 nt |
| **CtgVirga-1** | *Beet virus Q/Virgaviridae* | F - CTGTAAACGGCGATAGGATATACAGGC  R - GCTTCAGCGTAATCCTGTCAAATGGG | | 1936 nt |
| **CtgVirga-2** | *Chinese wheat mosaic virus/Virgaviridae* | F - AAATCTGGTGCACCCGACACGATGTT  R - AAGTTGATGGTGCCTCGTGTACGAC | | 384 nt |

^1^ F, forward primer; R, reverse primer

**Table S2.** Accession numbers of the reference sequences used in the phylogenetic analysis.

| **Name** | **Abbreviation** | **Family** | **Accession no.** | **^1^ Additional information** |
| --- | --- | --- | --- | --- |
| *Citrus tristeza virus* | CTV | *Closteroviridae* | GQ454869 | HA18-9 isolate |
| *Citrus tristeza virus* | CTV | *Closteroviridae* | JX266712 | Taiwan-Pum/SP/T1 isolate |
| *Citrus tristeza virus* | CTV | *Closteroviridae* | FJ525435 | NZRB-M17 isolate |
| *Citrus tristeza virus* | CTV | *Closteroviridae* | FJ525432 | NZRB-G90 isolate |
| *Citrus tristeza virus* | CTV | *Closteroviridae* | JF957196 | B301 isolate |
| *Citrus tristeza virus* | CTV | *Closteroviridae* | FJ525434 | NZRB-TH30 isolate |
| *Citrus tristeza virus* | CTV | *Closteroviridae* | FJ525433 | NZRB-TH28 isolate |
| *Citrus tristeza virus* | CTV | *Closteroviridae* | FJ525431 | NZRB-M12 isolate |
| *Citrus tristeza virus* | CTV | *Closteroviridae* | DQ272579 | Severe_Mexico isolate |
| *Citrus tristeza virus* | CTV | *Closteroviridae* | EU937521 | T36FS2-2_Florida isolate |
| *Citrus tristeza virus* | CTV | *Closteroviridae* | AY170468 | T36IC_Florida isolate |
| *Citrus tristeza virus* | CTV | *Closteroviridae* | AY340974 | Qaha_Egypt isolate |
| *Citrus tristeza virus* | CTV | *Closteroviridae* | JQ798289 | A18_Thailand isolate |
| *Citrus tristeza virus* | CTV | *Closteroviridae* | DQ151548 | T318A_Spain isolate |
| *Citrus tristeza virus* | CTV | *Closteroviridae* | JQ911664 | CT11A_China isolate |
| *Citrus tristeza virus* | CTV | *Closteroviridae* | AB046398 | Nuaga_Japan isolate |
| *Citrus tristeza virus* | CTV | *Closteroviridae* | JQ061137 | AT-1_China isolate |
| *Citrus tristeza virus* | CTV | *Closteroviridae* | KC748392 | SG29_Italy isolate |
| *Citrus tristeza virus* | CTV | *Closteroviridae* | EU937519 | VT_Israel isolate |
| *Citrus tristeza virus* | CTV | *Closteroviridae* | HM573451 | Kpg3_India isolate |
| *Citrus tristeza virus* | CTV | *Closteroviridae* | EU857538 | NZM16_SP isolate |
| *Citrus tristeza virus* | CTV | *Closteroviridae* | JQ911663 | CT14A_China isolate |
| *Citrus tristeza virus* | CTV | *Closteroviridae* | AF260651 | T30_Florida isolate |
| *Citrus tristeza virus* | CTV | *Closteroviridae* | Y18420 | T385_Spain isolate |
| *Citrus tristeza virus* | CTV | *Closteroviridae* | KC748391 | Bau282_Italy isolate |
| *Citrus tristeza virus* | CTV | *Closteroviridae* | AF001623 | SY568_California isolate |
| *Citrus tristeza virus* | CTV | *Closteroviridae* | JQ965169 | T68-1_Florida isolate |
| *Citrus tristeza virus* | CTV | *Closteroviridae* | FJ525436 | NZ-B18 isolate |
| *Citrus tristeza virus* | CTV | *Closteroviridae* | EU076703 | B165_India isolate |
| *Citrus tristeza virus* | CTV | *Closteroviridae* | JX266713 | Taiwan-Pum/M/T5 isolate |
| *Citrus tristeza virus* | CTV | *Closteroviridae* | GQ454870 | HA16-5_Hawaii isolate |
| *Citrus tristeza virus* | CTV | *Closteroviridae* | this study | CTV_SPBR_01 _this study |
| *Citrus tristeza virus* | CTV | *Closteroviridae* | this study | CTV_SPBR_02 _this study |
| *Citrus sudden death-associated virus* | CSDaV | *Tymoviridae* | DQ185573 | P15 isolate |
| *Citrus sudden death-associated virus* | CSDaV | *Tymoviridae* | AY884005 | CSDaV_1 |
| *Turnip yellow mosaic virus* | TYMV | *Tymoviridae* | NC_004063 | NAI |
| *Grapevine fleck virus* | GFkV | *Tymoviridae* | NC_003347 | NAI |
| *Maize rayado fino virus* | MRFV | *Tymoviridae* | NC_002786 | NAI |
| *Oat blue dwarf virus* | OBDV | *Tymoviridae* | U87832 | NAI |
| *Soybean chlorotic mottle virus* | SoyCMV | *Caulimoviridae* | NP_068729 | NAI |
| *Peanut chlorotic streak virus* | PCISV | *Caulimoviridae* | NP_042513 | NAI |
| *Figwort mosaic virus* | FMV | *Caulimoviridae* | NP_619548 | NAI |
| *Dahlia mosaic virus* | DMV | *Caulimoviridae* | AGT41978 | NAI |
| *Carnation etched ring virus* | CERV | *Caulimoviridae* | NP_612577 | NAI |
| *Cauliflower mosaic virus* | CaMV | *Caulimoviridae* | AAA46358 | NAI |
| *Strawberry vein banding virus* | SVBV | *Caulimoviridae* | AKB94071 | NAI |
| *Citrus endogenous pararetrovirus* | CitPRV | *Caulimoviridae* | NC_023153 | NAI |
| *Aristotelia chilensis virus 1* | AcV1 | *Caulimoviridae* | AHN13810 | NAI |
| *Petunia vein clearing virus* | PVCV | *Caulimoviridae* | AAK68664 | NAI |
| *Cassava vein mosaic virus* | CsVMV | *Caulimoviridae* | AAA79873 | NAI |
| *Cacao swollen shoot virus* | CSSV | *Caulimoviridae* | CAE81279 | NAI |
| *Commelina yellow mottle virus* | CoYMV | *Caulimoviridae* | CAA37110 | NAI |
| *Sugarcane bacilliform MO virus* | SCBV | *Caulimoviridae* | YP_595725 | NAI |
| *Rice tungro bacilliform virus* | RTBV | *Caulimoviridae* | AAL55651 | NAI |
| *Saccharomyces cerevisiae (TY3-2)* |  | *Saccharomycetaceae* | CAA86713 | NAI |
| *Pestivirus reindeer-1 V60-Krefeld* | V60-Krefeld | *Flaviviridae* | AAF02524 | NAI |
| *Border disease virus* | BDV | *Flaviviridae* | NP_777541 | NAI |
| *Bovine viral diarrhea virus 1* | BVDV-1 | *Flaviviridae* | AKQ44350 | NAI |
| *Pronghorn antelope pestivirus* | Pronghorn pestivirus | *Flaviviridae* | YP_009026415 | NAI |
| *Porcine pestivirus* | Pestivirus Bungowannah | *Flaviviridae* | YP_008992092 | NAI |
| *Xinzhou spider virus 2* | XZSV2 | *Flaviviridae* | YP_009179222 | NAI |
| *Wuhan centipede virus* | WHCeV | *Flaviviridae* | YP_009254745 | NAI |
| *Wenling shark virus* | WLSV | *Flaviviridae* | YP_009179227 | NAI |
| *Wuhan flea virus* | WHFV | *Flaviviridae* | YP_009179404 | NAI |
| *Wuhan aphid virus 2* | WHAV2 | *Flaviviridae* | YP_009179379 | NAI |
| *Wuhan aphid virus 1* | WHAV1 | *Flaviviridae* | YP_009179389 | NAI |
| *Shuangao insect virus 7* | SAIV7 | *Flaviviridae* | YP_009179402 | NAI |
| *Wuhan cricket virus* | WHCV | *Flaviviridae* | YP_009179400 | NAI |
| *Bole tick virus 4* | BLTV4 | *Flaviviridae* | YP_009179221 | NAI |
| *Diaphorina citri flavi-like virus* | DcFLV | *Flaviviridae* | YP_009259672 | NAI |
| *Gamboa mosquito virus* | GMV | *Flaviviridae* | YP_009179224 | NAI |
| *Soybean cyst nematode virus 5* | SbCNV-5 | *Flaviviridae* | YP_009028573 | NAI |
| *Nakiwogo virus* |  | *Flaviviridae* | YP_009268608 | NAI |
| *Tacheng tick virus 8* | TCTV8 | *Flaviviridae* | YP_009179217 | NAI |
| *Macrosiphum euphorbiae virus 1* | MeV-1 | *Flaviviridae* | YP_009175071 | NAI |
| *Shuangao lacewing virus 2* | SALV2 | *Flaviviridae* | YP_009179223 | NAI |
| *Xingshan cricket virus* | XSCV | *Flaviviridae* | YP_009179220 | NAI |
| *Gentian Kobu-sho-associated virus* | GKaV | *Flaviviridae* | YP_007438864 | NAI |
| *Beihai barnacle viurs 1* | BHBV1 | *Flaviviridae* | YP_009179226 | NAI |
| *Shayang spider virus 4* | SYSV4 | *Flaviviridae* | YP_009179219 | NAI |
| *West Nile virus* | WNV | *Flaviviridae* | AAV54504 | NAI |
| *Jingmen tick virus* | JMTV | *putative Flaviviridae* | YP_009030000 | NAI |
| *Citrus jingmen-like virus* | CJLV | *Flaviviridae* | this study | CtgFlavi-1 contig |
| *Chinese wheat mosaic virus* | CWMV | *Virgaviridae* | BAP90385 | NAI |
| *Oat golden stripe virus* | OGSV | *Virgaviridae* | CAB57882 | NAI |
| *Sorghum chlorotic spot virus* | SrCSV | *Virgaviridae* | NP_659019 | NAI |
| *Beet soil-borne virus* | BSBV | *Virgaviridae* | ACS14040 | NAI |
| *Beet virus Q* | BVQ | *Virgaviridae* | NP_612605 | NAI |
| *Broad bean necrosis virus* | BBNV | *Virgaviridae* | NP_740760 | NAI |
| *Barley stripe mosaic virus* | BSMV | *Virgaviridae* | AAA79146 | NAI |
| *Poa semilatent virus* | PSLV | *Virgaviridae* | CAA86473 | NAI |
| *Lychnis ringspot virus* | LRSV | *Virgaviridae* | CAA86599 | NAI |
| *Indian peanut clump virus* | IPCV | *Virgaviridae* | NP_835282 | NAI |
| *Peanut clump virus* | PCV | *Virgaviridae* | NP_620047 | NAI |
| *Pea early-browning virus* | PEBV | *Virgaviridae* | NP_049325 | NAI |
| *Pepper ringspot virus* | PepRSV | *Virgaviridae* | NP_620033 | NAI |
| *Tobacco rattle virus* | TRV | *Virgaviridae* | ACX54058 | NAI |
| *Ribgrass mosaic virus* | RMV | *Virgaviridae* | ACV13194 | NAI |
| *Turnip vein-clearing virus* | TVCV | *Virgaviridae* | NP_046151 | NAI |
| *Wasabi mottle virus* | WMoV | *Virgaviridae* | AHW98777 | NAI |
| *Youcai mosaic virus* | YMoV | *Virgaviridae* | BAN15047 | NAI |
| *Odontoglossum ringspot virus* | ORSV | *Virgaviridae* | AAB49498 | NAI |
| *Streptocarpus flower break virus* | SFBV | *Virgaviridae* | YP_762617 | NAI |
| *Tobacco mild green mosaic virus* | TMGMV | *Virgaviridae* | NP_062913 | NAI |
| *Rehmannia mosaic virus* | ReMV | *Virgaviridae* | ALP75636 | NAI |
| *Tomato mosaic virus* | ToMV | *Virgaviridae* | CAD10425 | NAI |
| *Brugmansia mild mottle virus* | BruMMV | *Virgaviridae* | YP_001974323 | NAI |
| *Obuda pepper virus* | ObPV | *Virgaviridae* | NP_620841 | NAI |
| *Paprika mild mottle virus* | PaMMV | *Virgaviridae* | NP_671718 | NAI |
| *Hibiscus latent Fort Pierce virus* | HLFPV | *Virgaviridae* | BAP76306 | NAI |
| *Hibiscus latent Singapore virus* | HLSV | *Virgaviridae* | YP_719997 | NAI |
| *Kyuri green mottle mosaic virus* | KGMMV | *Virgaviridae* | NP_619684 | NAI |
| *Zucchini green mottle mosaic virus* | ZGMMV | *Virgaviridae* | NP_624336 | NAI |
| *Cucumber fruit mottle mosaic virus* | CFMMV | *Virgaviridae* | AEV40683 | NAI |
| *Cucumber green mottle mosaic virus* | CGMMV | *Virgaviridae* | BAA18895 | NAI |
| *Sunn-hemp mosaic virus* | SHMV | *Virgaviridae* | P89202 | NAI |
| *Frangipani mosaic virus* | FrMV | *Virgaviridae* | AEW67306 | NAI |
| *Citrus virga-like virus* | CVLV | *Virgaviridae* | this study | CtgVirga-1 contig |
| *Citrus virga-like virus* | CVLV | *Virgaviridae* | this study | CtgVirga-2 contig |

^1^ NAI, no additional information

**Table S3.** Query coverage and maximum amino acid identity obtained from the BLASTx analysis against to the viral database and using the assembled viral contigs from this work as query sequences. Putative virus encoded protein and E value are shown.

| **Closely related species** | **Contig. length used as query (nt)** | **Query % coverage** | **Maximum % identity** | **E-value** | **Putative virus encoded protein** |
| --- | --- | --- | --- | --- | --- |
| *Citrus tristeza virus* | 3180 | 99 | 92 | 0.0 | Polyprotein replicase |
| *Citrus sudden death-associated virus* | 6109 | 97 | 98 | 0.0 | Polyprotein |
| *Marine RNA virus SF-2* | 1400 | 50 | 22 | 0.019 | Coat protein |
| *Rice stripe necrosis virus* | 250 | 79 | 39 | 1,00E-06 | Replication protein |
| *Rhizoctonia solani negative-stranded virus 4* | 126 | 97 | 56 | 3,00E-10 | RdRP |
| *Norovirus cat* | 144 | 64 | 48 | 0.026 | RdRP |
| *Dioscorea bacilliform AL virus* | 140 | 24 | 43 | 3.3 | RT-like |
| *Po-Circo-like virus 51* | 305 | 54 | 43 | 0.046 | Replicase |
| *Aphid lethal paralysis virus* | 343 | 99 | 97 | 3,00E-70 | Nonstructural protein |
| *Nakiwogo virus* | 2512 | 21 | 27 | 0.001 | Nonstructural protein NS3 |
| *Sclerotinia sclerotiorum deltaflexivirus 1* | 329 | 88 | 62 | 3,00E-34 | Polyprotein |
| *Soybean leaf-associated mycoflexivirus 1* | 196 | 85 | 37 | 0.051 | RdRP |
| *Deformed wing virus* | 173 | 53 | 52 | 0.010 | Polyprotein |
| *Nilaparvata lugens honeydew virus-3* | 118 | 96 | 47 | 7,00E-04 | RT-like |
| *Raphanus sativus cryptic virus 1* | 183 | 67 | 41 | 0.001 | Coat protein |
| *Passerivirus A1* | 134 | 85 | 46 | 0.42 | Polyprotein |
| *Chilli veinal mottle virus* | 109 | 96 | 48 | 0.016 | Polyprotein |
| *Citrus endogenous pararetrovirus* | 3339 | 53 | 72 | 0.0 | Polyprotein |
| *Lettuce necrotic leaf curl virus* | 141 | 80 | 42 | 0.11 | Polyprotein |
| *Rice tungro spherical virus* | 189 | 95 | 43 | 0.028 | RdRP |
| *Fusarium graminearum deltaflexivirus 1* | 262 | 98 | 71 | 4,00E-36 | Replication protein |
| *Boutonnet virus* | 423 | 60 | 36 | 2,00E-06 | Polyprotein |
| *Bufivirus UC1* | 203 | 93 | 43 | 5,00E-10 | RT-like |
| *Fisavirus 1* | 101 | 95 | 56 | 0.020 | Polyprotein |
| *Twyford virus* | 186 | 72 | 44 | 0.054 | Helicase |
| *Beet virus Q* | 4097 | 18 | 33 | 6,00E-25 | Replication protein |
| *Chinese wheat mosaic virus* | 2626 | 52 | 28 | 9,00E-29 | Replicase readthrough |

nt, nucleotide
